# Unraveling the Health Effects of Environmental Mixtures: An NIEHS Priority

**DOI:** 10.1289/ehp.1206182

**Published:** 2013-01-01

**Authors:** Danielle J. Carlin, Cynthia V. Rider, Rick Woychik, Linda S. Birnbaum

**Affiliations:** National Institute of Environmental Health Sciences, National Institutes of Health, Department of Health and Human Services, Research Triangle Park, North Carolina, E-mail: danielle.carlin@nih.gov

Traditionally, toxicological studies and human health risk assessments have focused primarily on single chemicals. However, people are exposed to a myriad of chemical and nonchemical stressors every day and throughout their lifetime. Some recent events that highlight the need to understand these complex exposures and their role in the etiology of disease include the *Deepwater Horizon* oil spill, the earthquake in Japan and subsequent Fukushima nuclear disaster, and the unknown environmental and health effects of hydraulic fracturing. Additionally, nonchemical stressors such as infectious agents, diet, and psychosocial stress should be examined for their contribution to health effects associated with chemical exposures. It is imperative to develop methods to assess the health effects associated with complex exposures in order to minimize their impact on the development of disease.The National Institute of Environmental Health Sciences (NIEHS) has a rich background in both supporting and conducting combined exposure research, and this topic will continue to be a priority at the institute.

The NIEHS has just completed its 2012–2017 Strategic Plan (NIEHS 2012c), which will guide the research priorities of the institute over the next 5 years (Birnbaum 2012). One of the goals of the Strategic Plan expressly addresses combined exposures. Concomitant with the Strategic Plan development process, the NIEHS conducted a workshop titled “Advancing Research on Mixtures: New Perspectives and Approaches for Predicting Adverse Human Health Effects” held 26–27 September 2011 in Chapel Hill, North Carolina. The workshop brought together experts from diverse disciplines (statistics, toxicology, epidemiology, risk assessment, and exposure science) to identify knowledge gaps, prioritize research areas, and recommend research strategies to address specific topics in the field of mixtures science. Here, we highlight findings from the workshop and put them in the context of NIEHS research efforts in mixtures.

NIEHS has a long history of intramural and extramural effort in mixtures research spanning at least three decades, including studies of a broad range of mixtures (Birnbaum et al. 1983; Suk et al. 2002; Yang and Rauckman 1987). The first NIEHS dedicated mixtures grants were funded in 1998 as a response to the Request for Applications (RFA) titled “Chemical Mixtures in Environmental Health” (NIEHS 1997). Another avenue for mixtures research has been the Superfund Research Program (NIEHS 2012d), which continues to support research related to Superfund sites that inherently contain multiple contaminants. Some of the topics covered by NIEHS grantees include air pollution and particulate matter, polybrominated diphenyl ethers (PBDEs), polyaromatic hydrocarbons (PAHs), pesticides, metals, and endocrine disruptors.

The NIEHS’s intramural research efforts have included investigation of the *Deepwater Horizon* oil spill. The Gulf Long-Term Follow-Up (GuLF) Study is evaluating the health effects, including biochemical and physiological effects from exposure to chemical and nonchemical stressors over an extended period (NIEHS 2012b). Intramural research in the Division of the National Toxicology Program (DNTP) has included work to assess the toxic equivalency factor approach used with dioxin-like compounds (Walker et al. 2005), complex mixtures (e.g., herbals, flame retardants, welding fumes), defined mixtures (e.g., 25-chemical mixture of groundwater contaminants, AIDS combination therapies), and use of high throughput technologies to address mixtures questions.

The NIEHS Mixtures Workshop presented an opportunity to involve the research community interested in combined exposures in a discussion of the state of the science and prioritization of research goals. Experts from many disciplines (risk assessment, exposure science, biology/toxicology, epidemiology, and statistics) convened to discuss current knowledge gaps in mixtures research and to develop suggested research priorities. The first day of the workshop consisted of presentations from speakers in each discipline on the current understanding and major challenges associated with mixtures research. Following these presentations, discipline-specific groups met to develop and prioritize a list of important knowledge gaps and research topics. The second day of the workshop involved presentations on novel perspectives for addressing mixtures challenges, such as cross-discipline experimental design considerations, environment-wide association studies (EWAS), and multipollutant epidemiological approaches. These presentations were followed by multidisciplinary breakout sessions in which a key topic and priority matrix scheme (consisting of time frame and scientific impact) was provided to the groups for discussion. The groups were also tasked with proposing suggested approaches to evaluate the most highly ranked topics.

A comprehensive report from the workshop contains presentation summaries, discussion, and breakout session results (NIEHS 2012a) along with background materials, slides from presenters, and summary presentations of breakout sessions.

The NIEHS Mixtures Workshop and the strategic planning process coincided with many other mixtures-related activities (e.g., the workshop on mixtures held 27–28 July 2011 in Washington, DC, by the National Academy of Sciences Standing Committee on the Use of Emerging Science for Environmental Health Decisions; the International Toxicology of Mixtures Conference held 21–23 October 2001 in Arlington, VA). Several common themes emerged from these mixtures-related events that were consistent with the findings from the NIEHS Mixtures Workshop. These themes, described below, will provide future directions and priority research areas to elucidate the health effects associated with combined exposures.

The first theme involves increasing our understanding of complex exposures, which will require efforts in three distinct yet interrelated research areas: *a*) monitoring, *b*) modeling, and *c*) developing unbiased approaches for characterizing exposures (e.g., exposome). Monitoring efforts require development of improved tools to capture everyday exposures as well as exposures over time. Examples include subdermal microchips or other portable monitoring devices, such as cell phones, to provide real-time, robust data on exposures. Data from monitoring devices, along with data from geographical information systems, will help in the development and validation of modeling approaches for estimating exposures. Modeling tools, such as software designed to mimic real-world exposures, will be especially helpful in understanding exposure over time, as well as population-level exposures. Last, unbiased approaches (i.e., approaches that provide comprehensive characterization of exposure without an *a priori* hypothesis) such as the exposome are needed to move beyond “looking under the lamppost” (i.e., only measuring exposure for chemicals with available toxicology data). The exposome is a tool that allows for characterization of exposure from all routes, all sources, and all chemicals, whether they are endogenous or exogenous (Rappaport and Smith 2010). The exposome approach also touches upon the challenge of internal versus external exposure. In terms of mixtures, the critical question is whether or not the mixtures that reside in the environment are the same mixtures that affect the cells in the body.

A second theme involves the prioritization of mixtures for study. Although the number of potential chemical combinations is practically infinite, humans are not equally likely to be exposed to every possible combination. Several methods are available or under development for narrowing the field of potential combinations for study. For example, databases, such as the National Health and Nutrition Examination Survey (NHANES) database, could be exploited to determine which combinations of measured chemicals represent common exposures in humans. Another proposed approach is the EWAS approach, which uses genomics techniques to query associations between all possible environmental exposures and particular disease end points or biomarkers (Patel et al. 2010). The maximum cumulative ratio approach has been suggested as a tool for prioritizing chemical combinations for cumulative risk assessment (Price and Han 2011). Finally, high throughput screening (HTS) approaches [e.g., Tox21 (U.S. Environmental Protection Agency 2012)] could be harnessed to rapidly evaluate a large number of chemical combinations to identify interactions (Shukla et al. 2010). Once interactions are identified, those chemical combinations could be tagged for further assessment *in vivo*. However, before HTS can be confidently applied to mixtures, additional chemistry requirements associated with multiple chemicals in a small volume must be addressed. In addition, it is critical to link *in vitro* assays and results to biologically meaningful end points that have been validated *in vivo*. Mixtures studies that incorporate both *in vitro* and *in vivo* components are needed to build these bridges.

Another theme that emerged was the need to apply a systems biology approach to mixtures research. To accomplish this goal, it is necessary to increase our understanding of the underlying pathways associated with disease. This theme relates to the movement in toxicology from a chemical focus to a pathway or network-disruption focus. This is especially important in mixtures research because a pathway focus would allow us to potentially predict interactions of chemicals that target a common pathway or system without testing all potential chemical combinations.

A common theme throughout the various events was the need for increased cross-disciplinary training and collaboration. Specifically, there is a need for further collaboration among epidemiologists, toxicologists, and biostatisticians to strengthen the associations identified in epidemiological studies. Examples of opportunities for cross-disciplinary collaboration include application of relative potency factors generated in toxicology studies to epidemiological assessments and use of epidemiological findings to identify important combinations for toxicological studies (e.g., using epidemiological data that suggests interactions between chemicals). Another area that requires collaboration is the development of better statistical methods for assessing the effects of multipollutant exposures in epidemiological studies. Overall, mixtures studies require novel and sophisticated mathematical, statistical, computational, and analytical tools, which will be dependent on continuous collaboration among the various disciplines. The uncertainty in the cumulative risk assessment process will be decreased through development and improvement of predictive models of mixture toxicity, such as component-based or sufficient similarity of whole mixtures (Rice et al. 2009).

Last, participants in the NIEHS Mixtures Workshop discussed the importance of developing mixtures-related databases. Because it is challenging to search the literature for interactions among chemicals, there was a great deal of interest in the development of databases that would accommodate different levels of mixture study results (i.e., *in vitro* studies to human studies). In addition, there is a need for standardization and integration across data sets, and user-friendly bioinformatics tools and interfaces are also needed so researchers can effectively use available data. Establishing the scope and developing an implementation strategy will require significant planning.

The field of mixtures research has evolved from evaluating simple combinations of chemicals in search of interactions to considering the prioritization of mixtures for study based on exposure and systems biology. In parallel, human health risk assessment has moved from a chemical-by-chemical approach to conducting cumulative risk assessments and community-based risk assessments that attempt to capture the totality of chemical and nonchemical stressors of a given population. However, we still need to define real-world exposures, increase our ability to link those exposures to the development of disease, and find answers to many other questions. The NIEHS, through its extramural and intramural efforts, will utilize the results from the NIEHS Mixtures Workshop and the strategic planning process by incorporating the priority study areas listed above into both ongoing and future mixtures research projects, and foster collaborations between the various disciplines through workshop interactions and translational activities.

## References

[r1] Birnbaum LS (2012). NIEHS’s new strategic plan.. Environ Health Perspect.

[r2] Birnbaum LS, Darcey DJ, McKinney JD (1983). Hexabromonaphthalene contaminants of polybrominated biphenyls: chemical composition and disposition in the rat.. J Toxicol Environ Health.

[r3] NIEHS (National Institute of Environmental Health Sciences). 1997. Chemical Mixtures in Environmental Health. RFA-ES-98-002. Available: http://grants.nih.gov/grants/guide/rfa-files/RFA-ES-98-002.html [accessed 7 December 2012].

[r4] NIEHS (National Institute of Environmental Health Sciences). 2012a. Advancing Research on Mixtures: New Perspectives and Approaches for Predicting Adverse Human Health Effects. Workshop Summary. Available: http://www.niehs.nih.gov/about/visiting/events/pastmtg/2011/mixtures/pdf_workshop_report.pdf [accessed 7 December 2012].

[r5] NIEHS (National Institute of Environmental Health Sciences). 2012b. Gulf Study Homepage.Available: http://www.niehs.nih.gov/about/od/programs/gulfspill/gulfstudy/index.cfm [accessed 7 December 2012].

[r6] NIEHS (National Institute of Environmental Health Sciences). 2012c. NIEHS Strategic Plan. Available: http://www.niehs.nih.gov/about/strategicplan/index.cfm [accessed 7 December 2012].

[r7] NIEHS (National Institute of Environmental Health Sciences). 2012d. Superfund Research Program Homepage. Available: http://www.niehs.nih.gov/research/supported/srp/ [accessed 7 December 2012].

[r8] Patel CJ Bhattacharya J Butte AJ 2010 An environment-wide association study (EWAS) on type 2 diabetes mellitus. PLoS One 5 5 e10746; doi: [Online 20 May 2010] 10.1371/journal.pone.0010746 20505766 PMC2873978

[r9] Price PS, Han XL (2011). Maximum cumulative ratio (MCR) as a tool for assessing the value of performing a cumulative risk assessment.. Int J Environ Res Public Health.

[r10] Rappaport SM, Smith MT (2010). Environment and disease risks.. Science.

[r11] Rice GE, Teuschler LK, Bull RJ, Simmons JE, Feder PI (2009). Evaluating the similarity of complex drinking-water disinfection by-product mixtures: overview of the issues.. J Toxicol Environ Health A.

[r12] Shukla SJ, Huang R, Austin CP, Xia M (2010). The future of toxicity testing: a focus on *in vitro* methods using a quantitative high-throughput screening platform.. Drug Discov Today.

[r13] Suk WA, Olden K, Yang RS (2002). Chemical mixtures research: significance and future perspectives.. Environ Health Perspect.

[r14] U.S. Environmental Protection Agency. 2012. Tox21 Homepage. Available: http://epa.gov/ncct/Tox21/ [accessed 7 December 2012].

[r15] Walker NJ, Crockett PW, Nyska A, Brix AE, Jokinen MP, Sells DM (2005). Dose-additive carcinogenicity of a defined mixture of “dioxin-like compounds.”. Environ Health Perspect.

[r16] Yang RS, Rauckman EJ (1987). Toxicological studies of chemical mixtures of environmental concern at the National Toxicology Program: health effects of groundwater contaminants.. Toxicology.

